# Effect of the Human Amniotic Membrane on the Umbilical Vein Endothelial Cells of Gestational Diabetic Mothers: New Insight on Inflammation and Angiogenesis

**DOI:** 10.3389/fbioe.2022.854845

**Published:** 2022-07-05

**Authors:** Caterina Pipino, Ángel Bernabé-García, Ilaria Cappellacci, Javier Stelling-Férez, Pamela Di Tomo, Manuela Santalucia, Carlos Navalón, Assunta Pandolfi, Francisco José Nicolás

**Affiliations:** ^1^ Center for Advanced Studies and Technology-CAST (ex CeSI-MeT), Department of Medical, Oral and Biotechnological Sciences, University G. D’Annunzio Chieti-Pescara, StemTeCh Group, Chieti, Italy; ^2^ Regeneration, Molecular Oncology and TGFß, IMIB-Arrixaca, Hospital Clínico Universitario Virgen de La Arrixaca, Murcia, Spain; ^3^ Department of Nutrition and Food Technology, UCAM, Murcia, Spain

**Keywords:** amniotic membrane (AM), HUVECs, diabetes, wound healing, inflammation, angiogenesis

## Abstract

One of the most relevant diabetes complications is impaired wound healing, mainly characterized by reduced peripheral blood flow and diminished neovascularization together with increased inflammation and oxidative stress. Unfortunately, effective therapies are currently lacking. Recently, the amniotic membrane (AM) has shown promising results in wound management. Here, the potential role of AM on endothelial cells isolated from the umbilical cord vein of gestational diabetes-affected women (GD-HUVECs), has been investigated. Indeed, GD-HUVECs *in vivo* exposed to chronic hyperglycemia during pregnancy compared to control cells (C-HUVECs) have shown molecular modifications of cellular homeostasis ultimately impacting oxidative and nitro-oxidative stress, inflammatory phenotype, nitric oxide (NO) synthesis, and bioavailability, thus representing a useful model for studying the mechanisms potentially supporting the role of AM in chronic non-healing wounds. In this study, the anti-inflammatory properties of AM have been assessed using a monocyte–endothelium interaction assay in cells pre-stimulated with tumor necrosis factor-α (TNF-α) and through vascular adhesion molecule expression and membrane exposure, together with the AM impact on the nuclear factor kappa-light-chain-enhancer of activated B cell (NF-kB) pathway and NO bioavailability. Moreover, GD-HUVEC migration and tube formation ability were evaluated in the presence of AM. The results showed that AM significantly reduced TNF-α-stimulated monocyte–endothelium interaction and the membrane exposure of the endothelial vascular and intracellular adhesion molecules (VCAM-1 and ICAM-1, respectively) in both C- and GD-HUVECs. Strikingly, AM treatment significantly improved vessel formation in GD-HUVECs and cell migration in both C- and GD-HUVECs. These collective results suggest that AM positively affects various critical pathways in inflammation and angiogenesis, thus providing further validation for ongoing clinical trials in diabetic foot ulcers.

## Introduction

Hyperglycemia-induced endothelial dysfunction contributes to the development of inflammation and impaired angiogenesis in diabetes mellitus (Kolluru et al., 2012). Diabetic patients have lower ability to metabolize glucose resulting in hyperglycemic conditions making them more susceptible to inflammation and infection (Kolluru et al., 2012) which may contribute to diabetic wound impairment. It is estimated that 15% of patients with diabetes may develop foot ulcers, responsible for 50% of all diabetes-related hospitalization with negative effects on quality of life and a significant impact also on the healthcare economics (Ahmad, 2016). The formation of skin ulceration in a diabetic foot is a severe medical condition that, without correct healing, can lead to amputation (Bandyk, 2018). Many factors may contribute to impaired wound healing, among which are altered inflammatory response, decreased angiogenesis, progressive skin connective tissue atrophy, and oxidative stress (Tentolouris et al., 2021).

After wounding, endothelial cells are activated, a wide spectrum of cell surface adhesion molecules are overexpressed and leukocytes, neutrophils, and macrophages are recruited to perform the inflammatory and proliferative phases (Okonkwo and Dipietro, 2017). One of the major mediators of this event is the activation of nuclear factor kappa-light-chain-enhancer of activated B cell (NF-kB) pathway triggering, in turn, the upregulation of the expression of the vascular cell adhesion molecules (VCAM-1) and intercellular cell adhesion molecules (ICAM-1) in response to various inflammatory cytokines (Di Tomo et al., 2012).

In addition to inflammation, delayed healing of diabetic wounds is also characterized by impaired angiogenesis responses (Abaci et al., 1999; Waltenberger et al., 2000; Eelen et al., 2015) leading to chronic wounds due to a lack of preventive and adequate interventions. Moreover, nitric oxide (NO) plays a key role in regulating many aspects of inflammatory responses and angiogenesis (Wallace, 2005). Indeed, it is well known that NO is able, under normal conditions, to promote angiogenesis, migration, and proliferation of fibroblast, epithelial cells, endothelial cells, and keratinocytes (Efron et al., 2000). In diabetic foot ulcers (DFUs), hyperglycemia increases vascular super dioxide (O2-) production leading to NO inactivation that subsequently contributes to vascular dysfunction and foot ulcer development (Boykin, 2010).

Therefore, strategies to reduce inflammation and to simultaneously enhance neovascularization may dramatically improve the quality of life of these patients and significantly reduce the global biomedical burden. Nowadays, among recent DFU therapeutic applications, the amniotic membrane (AM) is one of the most promising ones (Zelen 2013; Valiente et al., 2018; Tettelbach et al., 2019; Doucette et al., 2020). AM, as a wound dressing, has been used for over a century and it is an ideal biological graft. AM is made up of a single epithelial cell layer, a thick basement membrane, and an avascular stroma (Silini et al., 2020). So far, many studies regarding the application of AM in DFU have been performed not only to test new possible applications but also to identify processing protocols (Lakmal et al., 2021). However, the role of AM and the mechanisms underlying inflammation and angiogenesis are not fully understood. Thus, to know the effect of AM on endothelial cells during AM treatment of DFUs would be desirable to understand its role in this condition.

Recently, the availability of cellular models representing endothelium affected by hyperglycemic conditions had become very useful to investigate the mechanisms of the disease and assay concrete therapeutic options. Interestingly, we have employed cultured endothelial cells that *in vivo* were exposed to chronic hyperglycemia during pregnancy (Di Fulvio et al., 2014).

Indeed, our previous results showed that these cells, isolated from the human umbilical cord veins of women affected by gestational diabetes (GD-HUVECs), exhibit epigenetic modifications leading to a durable pro-inflammatory phenotype potentially predisposing to endothelial dysfunction (Di Fulvio et al., 2014; Di Tomo et al., 2021). Thus, GD-HUVECs can be used as a valuable model for studying the diabetic endothelium typical of DFUs. Moreover, HUVECs have been commonly used to study molecular and signaling mechanisms related to angiogenesis, a wound repair fundamental process that involves endothelial cell activation, migration, and proliferation (Medina-Leyte et al., 2020).

In this study, by stimulating GD-HUVECs with a pro-inflammatory stimulus such as a low level of tumor necrosis factor-alpha (TNF-α), we have generated an environment like the one found in diabetic foot ulcer endothelium. Therefore, by using this model we have investigated mechanisms through which AM can affect inflammation, migration, and angiogenesis to better understand its positive role in the diabetic chronic wound environment.

## Materials and Methods

### HUVEC Cultures and Experimental Protocol

This study was carried out on HUVECs isolated from the umbilical cord veins of newborns delivered between the 36th and the 40th gestational week at the Hospital of Chieti and Pescara (Italy) from randomly selected mothers affected by gestational diabetes (GD) and healthy Caucasian mothers (control, C), according to previously published methods (Ucci et al., 2019). All procedures agreed with the ethical standards of the Institutional Committee on Human Experimentation (reference number 1879/09COET) and with the Declaration of Helsinki principles. The protocol was approved by the Institutional Review Board and informed consent was signed by each participating subject. Briefly, umbilical cords were collected immediately after delivery, then umbilical cord veins were cannulated and perfused with 1 mg/ml collagenase1A at 37°C and HUVECs collected in a endothelial growth medium (HUVEC medium) composed by DMEM/M199 (1:1) supplemented with 1% l-glutamine, 1% penicillin–streptomycin, and 20% fetal bovine serum (FBS). Then, the cell suspension was centrifuged at 1,200 rpm for 10 min and the cell pellet was resuspended in a HUVEC medium and plated on 1.5% gelatin-coated tissue culture plates. Primary C-HUVECs and GD-HUVECs were characterized as von Willebrand factor positive and alpha-smooth muscle cell actin negative; for all experiments, the cells were *in vitro* used between the 3rd and 5th passage.

Related to the amniotic membrane, the samples and data from patients included in this study, who gave the written informed consent, were collected, processed, and provided by the Biobanco en Red de la Región de Murcia, BIOBANC-MUR, registered on the Registro Nacional de Biobancos with registration number B.0000859, and were processed following the standard operating procedures with an appropriate approval of the Ethical and Scientific Committees.

HUVECs were grown on 1.5% gelatin-coated tissue culture plates (Sigma-Aldrich) in a complete low-glucose (1 g/L) DMEM and M199 medium (ratio 1:1) supplemented with 20% FBS, 10 μg/ml heparin and 50 μg/ml endothelial cell growth factor, 1% penicillin/streptomycin, and 1% l-glutamine (all from Biowest, Nuaillé, France) (standard medium).

All experiments were performed in technical triplicate using at least three different cellular strains (*n* = 3) of C- and GD-HUVECs following the experimental protocols illustrated in Supplementary Figure S1.

### AM Collection and Storage

The amniotic membrane was prepared as previously described (Ruiz-cañada et al., 2017). Briefly, the term placenta from healthy donor mothers, average age of 36, was obtained from uncomplicated cesarean section. The fetal membranes were washed in physiological saline solution (PSS) (B.Braun, Barcelona, Spain) supplemented with 50 μg/ml amphotericin (Bristol-MyersSquibb, Madrid, Spain), 48 μg/ml clotrimazole (Almirall-Prodesfarma, Barcelona, Spain), 50 μg/ml tobramycin (Laboratorios Normon, Madrid, Spain), and 50 μg/ml vancomycin (Laboratorios Hospira, Madrid, Spain) and rapidly transferred to the laboratory under sterile conditions. Under a laminar flow cabinet, the amnion was mechanically peeled from the chorion, washed three to four times with 200 ml of PBS (Biowest, Nuaillé, France), and flattened onto the sterile nitrocellulose paper (Pierce, Thermo Fisher Scientific, Waltham, MA United States) with the amniotic epithelium surfaced up, and the spongy layer facing and sticking to the nitrocellulose paper. Then, the paper with adhered membrane was cut into 1 cm × 1 cm fragments. Freshly cut AM fragments were separated from paper pieces and placed in a sterile vial containing a freezing solution made of 10% dimethyl sulfoxide (DMSO) (Sigma-Aldrich, St Louis, MO, United States), 4% human albumin (Grifols, Bercelona, Spain) in the DMEM (Biowest, Nuaillé, France) medium and then frozen at −80°C and later preserved in liquid nitrogen until further use. On the day of the experiment, AM pieces were thawed at 37°C, then the pieces were washed three times with DMEM, and placed at 37°C in a 7.5% CO_2_ incubator for 2 hours for the re-vitalization of the amniotic membrane cells. Then, the appropriate number of AM pieces, related to the number of cells/culture plate area, was used for the desired experiment. Briefly, for the use of the amniotic membrane, we established a ratio of number of pieces of amniotic membrane per cultivated cell surface. The AM (1 square centimeter pieces) was used following this rationale: one piece of AM in a well of a 24 well plate (cell migration and Matrigel tube formation assays), three pieces for a 6 well plate well (flow cytometry assay, Western blot, monocyte adhesion assay, and gene expression assay) or five to six pieces in a 5 cm diameter plate (immunofluorescence assay). For the experiment replicates, a different AM donor was employed each time.

### Ribonucleic Acid Isolation and Real-Time Polymerase Chain Reaction

The gene expression of adhesion molecules was performed in serum-starved cells (medium with 0.5% FBS) that had been incubated for 24 h with AM and then treated with TNF-α (1 ng/ml) for 2, 6, or 24 h. Following treatments, RNA was extracted using the RNeasy-mini system (Qiagen). Afterward, 1 µg of RNA was retro-transcribed using iScript reagents (Bio-Rad). The resulted cDNA was used to perform a quantitative real-time PCR (RT-PCR) using the SYBR premix ex Taq kit (Takara Bio Europe) according to the manufacturer’s instructions to analyze the inflammation-associated genes (Table 1). Each analysis was normalized with glyceraldehyde 3-phosphate dehydrogenase (*GAPDH*) gene expression according to the 2-ΔΔCt method. The experiment was carried out on three different strains for C-HUVECs and three different strains for GD-HUVECs, each in technical triplicate.

**TABLE 1:** Primers used for quantitative PCR.

Gene name (GeneCards^®^)/primer name	Primer sequence 5′–3′
*CCL2* Fw	AGA​CTA​ACC​AGA​AAC​ATC​C (Sigma KiCqStart)
*CCL2* Rev	ATTGATTGCATCTGGCTG (Sigma KiCqStart)
*GAPDH* Fwd	AGC​TCA​GGC​CTC​AAG​ACC​TT
*GAPDH* Rev	AAG​AAG​ATG​CGG​CTG​ACT​GT
*ICAM1* Fwd	ACCATCTACAGCTTTCCG (Sigma KiCqStart)
*ICAM1* Rev	TCACACTTCACTGTCACC (Sigma KiCqStart)
*SELE* Fwd	GAG​AAT​TCA​CCT​ACA​AGT​CC (Sigma KiCqStart)
*SELE* Rev	AGG​CTT​GAA​CAT​TTT​ACC​AC (Sigma KiCqStart)
*VCAM1* (mix Fwd/Rev)	Proprietary sequence (Qiagen QuantiTect**®**) QT00018347

CCL2, C-C motif chemokine ligand 2. GADPH, glyceraldehyde-3-phosphate dehydrogenase. ICAM1, intercellular adhesion molecule 1. SELE, Selectin E. VCAM1, vascular cell adhesion molecule 1.

### Immunofluorescence Assay and Confocal Laser Scanning Microscopy Analysis

Control HUVECs and GD-HUVECs were grown until they reached 50% confluence on round glass coverslips placed in 5 cm diameter Petri dishes, using a standard medium (20% FBS, as aforesaid). At this point, the cells were PBS-washed, and the medium was replaced by the reduced FBS medium to begin a 24-h serum-starvation period. At the same time, five to six –AM pieces (1 cm^2^) were placed over the cell-seeded glass coverslips floating on the culture medium. After 24 h, control coverslips were removed from the Petri dish and fixed with paraformaldehyde 4% (AppliChem GmbH, Darmstadt, Germany) in PBS (Biowest, Nuaillé, France) for 15 min. TNF-⍺ (1 ng/ml) was added to the remaining coverslips at the 1 ng/ml final concentration. The coverslips were removed from the medium and fixed (as aforesaid) at the indicated times. Immunofluorescence was carried out as previously described (Alcaraz et al., 2015), briefly after blocking, the cells were incubated for 1 h (RT) with the proper antibody diluted in blocking buffer, washed and further incubated with the appropriate fluorescent-conjugated secondary antibodies in combination with Alexa Fluor 594 or Alexa Fluor 468 conjugated phalloidin depending on the secondary antibody used for the antigen detection (Molecular Probes, Thermo Fisher Scientific, Waltham, MA, United States) and Hoechst 33258 (Fluka, Biochemika, Sigma-Aldrich, St Louis, MO, United States) for 30 min. Finally, the samples were examined, and representative images were taken using the confocal microscope Leica TCS SP8 MP (Leica, Wetzlar, Germany). The acquisition of images was performed using Leica Application Suite X (LAS X) software. The antibodies used were anti-NF-kB (Abcam, ab16502, Cambridge, United Kingdom) and Anti-paxillin (Santa Cruz Biotechnology, sc-365379 Heidelberg, Germany). For proper assessment and interpretation of NF-kB nuclear translocation intensity, the maximum intensity projection of 7 z-stacks was used. The experiment was carried out on three different strains for C-HUVECs and three different strains for GD-HUVECs, each in technical triplicate.

### Flow Cytometry Analysis

At the basal state and after stimulations, non-permeabilized cells were detached by 5 mM EDTA, washed, and re-suspended in 0.5% BSA solution. The cells were treated and incubated with anti-VCAM-1 PE conjugate (Santa Cruz Biotechnology, sc-13160, 1:100) and with anti-ICAM-1 FITC conjugate (Santa Cruz Biotechnology, sc-107, 1:100) as previously described (Ucci et al., 2019). One test tube for the basal state and one for the TNF-α-treated condition were incubated with anti-VCAM-PE and anti-ICAM-FITC isotypes (normal mouse IgG2a FITC-conjugated and normal mouse IgG1 PE-conjugated, Santa Cruz Biotechnology, 1:100) as negative controls. Flow cytometry analysis was performed on a BD FACS Canto II flow cytometer (BD Bioscences) and for each sample 1 × 10^4^ events were analyzed using FACSDiva v 6.1.3 (BD Biosciences) and FlowJo 8.3.3 software (Tree Star Inc., Ashland, United States). All the results are expressed as mean fluorescence intensity (MFI) ratio ±standard deviation (SD). Each value was calculated by dividing the MFI of positive events by the MFI of negative events (MFI of secondary antibody). The experiment was carried out on four different strains for C-HUVECs and four different strains for GD-HUVECs, each in technical triplicate.

### Monocyte-HUVEC Adhesion Assays

The adhesion assay was performed in C- and GD-HUVECs under the basal condition and after incubation for 24 h with AM. In detail, the cells were grown in six-well tissue culture plates (200.000 cells/well) and, when confluent, the cells were serum-starved (0.5% FBS), then stimulated with TNF-α (1 ng/ml) for 16 h, and finally AM was added for 24 h. Three pieces of 1 cm^2^ AM were added per well.

Monocytes (U937 cell lines, European Collection of Authenticated Cell Cultures, ECACC) were used to evaluate the adhesion to HUVEC monolayers as previously described (Di Tomo et al., 2015; Ucci et al., 2019). Briefly, the medium was removed from each HUVEC well, then the cells were gently washed with DMEM, and 1 million of monocyte suspension was added to each well and incubated on HUVECs for 20 min with gentle shaking at room temperature. Finally, monocyte suspension was collected and HUVECs were gently washed, then the adherent monocytes on HUVECs were fixed with paraformaldehyde 1%. To identify the number of adherent monocytes for each tested strain, 12 counts for every experimental condition were performed (employing at least 3 different randomly selected high-power fields, ×10 magnification) using a quadrant. For this experiment, four different strains for C-HUVECs and four different strains for GD-HUVECs were used.

### Western Blot

Control- and GD-HUVECs were seeded in 6 well plates using the standard medium (20% FBS). Then, the cells were PBS-washed, and the medium was replaced by 0.5% FBS to begin a 24-h serum-starvation period. At the same time, three AM pieces (1 cm^2^) were placed over the cell by floating on the culture medium. After 24 h, then TNF-⍺ (1 ng/ml) was added for 1 and 3 h. Briefly, for protein extraction, the cells were washed with cold PBS and lysed using RIPA buffer (Sigma-Aldrich) supplemented with phosphatase inhibitors (I and II) and protease inhibitors (all from Sigma-Aldrich). The protein concentration of the lysates was determined using the Qubit Protein Assay kit (Invitrogen). The membranes were blocked with 5% BSA, followed by immunoblotting with the primary antibody against rabbit anti-p-NF-kB p65 (Ser536) (^93^H1) (1:1000, Cell Signaling Technology), overnight at 4°C, followed by rabbit horseradish peroxidase-conjugated secondary antibody (1:5000) (Santa Cruz Biotechnology). Then, the membranes were stripped and immunoblotted with the primary antibody against rabbit anti-NF-kB p65 (1:1000, Cell Signaling Technology), followed by rabbit horseradish peroxidase-conjugated secondary antibody (1:5000) (Santa Cruz Biotechnology). The immune complexes were visualized by means of the ECL Plus detection reagent (Thermo Scientific), and data were processed and quantified using UVITEC Alliance software. Protein densities were divided by β-actin densities (mouse monoclonal anti-β-actin, 1:10000), and the resulting ratio was considered as an index of p-NF-kB expression in arbitrary units. The experiment was carried out on three different strains for C-HUVECs and three different strains for GD-HUVECs, each in technical triplicate.

### cGMP Determination

Intracellular cyclic 3′-5′ guanosine monophosphate (cGMP) levels were evaluated using a commercially available Elisa kit (Cayman Chemical) as previously shown (Di Tomo et al., 2012). The experiment was carried out on six different strains for C-HUVECs and six different strains for GD-HUVECs, each in technical triplicate.

### Matrigel Tube Formation Assay

Control- and GD-HUVECs were plated (2.5 × 10^4^ cells/well) in the growth factor–reduced basement membrane matrix gel, also called Matrigel (BD Biosciences), coated 96-well plates using the 10% FBS HUVEC culture medium. Preliminary experiments were conducted to identify the suitable number of cells per well and the best serum concentration. After plating, the cells were incubated for 20 min at 37°C inducing cellular adhesion to Matrigel and then, in the respective wells, AM pieces were added to floating in the culture medium. Six hours later representative photographs were acquired using an inverted microscope (Olympus). Images were processed using ImageJ software (Angiogenesis analyzer tool) to analyze angiogenic parameters (i.e., number of segments, meshes, and master junctions) in the basal state and following the amniotic membrane incubation in three different strains of C-HUVECs and three different strains of GD-HUVECs in technical triplicate.

### Wound Healing Scratch Assay

For the wound healing scratch assay, C- and GD-HUVECs were seeded on 24 well plates and grown for 2 days, then the growth medium was changed for complete DMEM with 1% FBS for 24 h before the wound healing scratch assay. The wound was made by scratching a line across the bottom of the dish on a confluent cell’s monolayer using a sterile p-200 pipette tip. The cells were rinsed very gently with PBS and then cultivated in the medium with 1% FBS with the addition of AM pieces floating in the culture medium. Pictures were taken at ×10 magnifications using an inverted microscope (Olympus). To quantify migration, the area of the gap obtained by scratching a line across the bottom of the dish was quantified using ImageJ software (http://rsbweb.nih.gov/ij/). After each treatment, the area of the same gap was measured again. The difference between the initial and final areas was calculated. That difference was represented in each treatment and bigger differences indicate better migration. The experiment was carried out on three different strains for C-HUVECs and three different strains for GD-HUVECs, each in technical triplicate.

### Statistical Analysis

The results are presented as the means ± standard deviation (SD) or standard error of the mean (SEM) of at least three different experiments using at least three different cellular strains (*n* = 3) both of C-HUVECs and GD-HUVECs. The ANOVA test followed by the Tukey’s multiple comparison test for *post hoc* comparisons was used to analyze the differences between C- and GD-HUVECs and between the different treatments. The significance was defined as a *p*-value less than 0.05.

## Results

### Amniotic Membrane Decreases the Expression of Genes Involved in Inflammation

The gene expression of several genes involved in inflammation was performed to evaluate the ability of AM in preventing TNF-α-triggered inflammatory response in endothelial cells.

**FIGURE 1:**
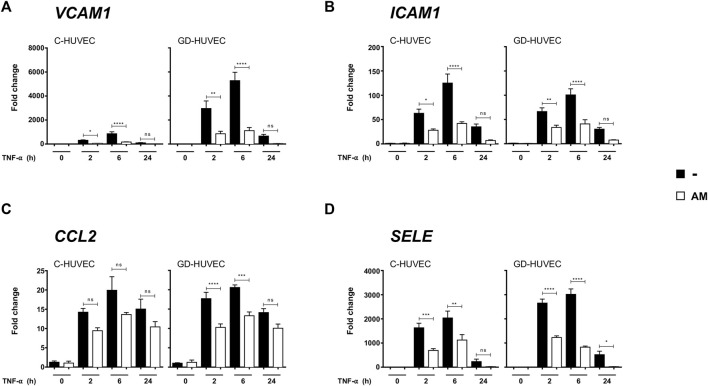
AM reduces the expression of genes involved in inflammation. Gene expression analysis of *VCAM-1* **(A)**, *ICAM-1* **(B)**, *CCL2* **(C)**, and *SELE* **(D)**, from C- and GD-HUVECs untreated and after TNF-α stimulation of cells that had been pretreated or not with AM for 24 h. Quantitative data in the histograms **(A–D)** show the mRNA relative expression (target gene expression normalized with GAPDH expression using the 2-ΔΔCt method). Each measurement is defined as the mean ± SD using three different strains for C-HUVECs and three different strains for GD-HUVECs (*n* = 3). ANOVA and Tukey’s multiple comparison test: *p < 0.05, **p < 0.01, ***p < 0.001 and ****p < 0.0001.

The cells, either C-HUVECs or GD-HUVECs, were first treated with AM for 24 h and then stimulated with TNF-α up to 24 h. The cells had a strong response to the TNF-α stimulation of all genes tested, and it was significant that *VCAM-1* and E-selectin (*SELE*) had a stronger response in GD-HUVECs compared to C-HUVECs (*p* < 0.001), for the rest of the genes, there was no apparent expression differences between C-HUVECs and GD-HUVECs (Figure 1). Strikingly, in all cases, a pronounced attenuation to the response to TNF-α was seen in samples previously treated with AM in both C-HUVECs and GD-HUVECs. Giving special attention results in the response to the expression of *VCAM-1* and *SELE* where the previous treatment with AM markedly attenuated the response to TNF-α (Figures 1A,D). All these data indicate that AM causes a decrease in the transcriptional response of HUVECs to TNF-α for the expression of these four genes involved in inflammation.

### Amniotic Membrane Reduces the Membrane Exposure of Endothelial Adhesion Molecules

**FIGURE 2:**
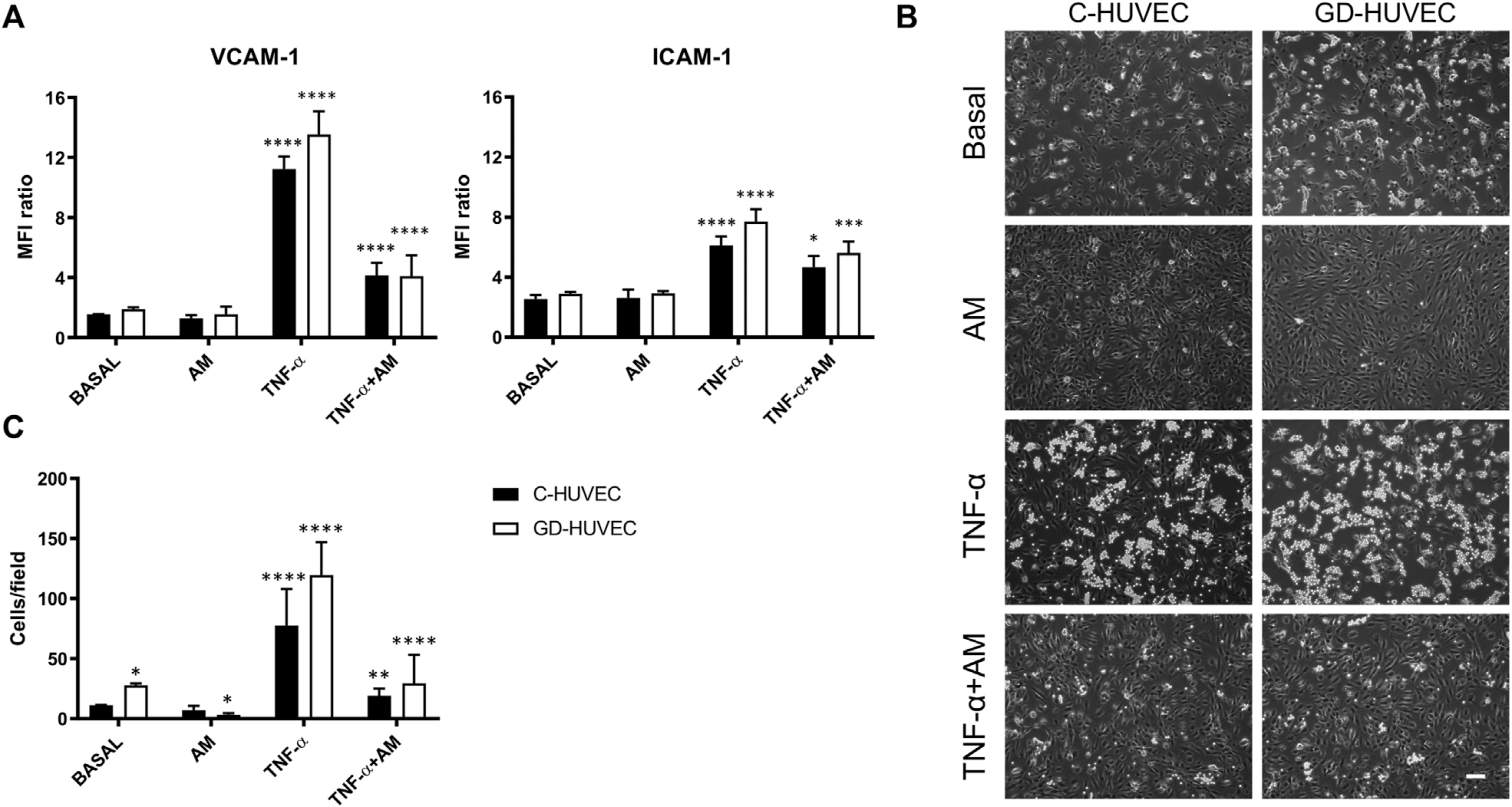
Effect of AM on VCAM-1 and ICAM-1 membrane exposure and TNF-α-induced monocyte adhesion in C- and GD-HUVECs analyzed by flow cytometry. **(A)** VCAM-1 and ICAM-1 membrane exposure in C- and GD-HUVECs untreated (basal) and treated for 16 h with 1 ng/ml of TNF-α with or without AM incubation (24 h). Quantitative data in the histograms show the mean fluorescence intensity (MFI) ratio between the signal and the background noise. The experiments were performed using five different cellular strains for C- and five different cellular strains for GD-HUVECs (*n* = 5). ANOVA and Tukey’s multiple comparison test: *p < 0.05 TNF-α + AM vs. TNF-α; ***p < 0.001 TNF-α+AM vs. TNF-α; ****p < 0.0001 TNF-α vs. basal and vs. AM; ****p < 0.0001 TNF-α + AM vs. TNF-α. **(B)** Monocyte-endothelial cell interaction on C- and GD-HUVECs untreated (basal) and incubated with AM (24 h) after TNF-α stimulation for 16 h. Representative pictures of C- and GD-HUVECs for each experimental condition. Bar represents 100 µm. Quantitative data, in the histogram **(C)**, show the number of adherent U937 cells obtained by analyzing the microscope images of four different cellular strains for both C-HUVECs and GD-HUVECs (*n* = 4), each including 12 counts per condition. Each measurement is defined as the mean ± SD of adherent monocytes. ANOVA and Tukey’s multiple comparison test: *p < 0.05 basal GD-HUVEC vs. basal C-HUVEC and AM GD-HUVEC vs. basal GD-HUVEC; **p < 0.01 TNF-α + AM vs. TNF-α; ****p < 0.0001 TNF-α vs. basal and vs. AM, TNF-α + AM vs. TNF-α.

To further validate these findings, VCAM-1 and ICAM-1 membrane exposure, the main mechanism behind the interaction between monocytes and endothelial cells, was evaluated. In order to reproduce the inflammatory milieu observed in DFUs, the amniotic membrane potential (24 h of incubation) was tested on C- and GD-HUVECs under basal conditions, as well as in cells previously treated with 1 ng/ml of TNF-α for 16 h. Figure 2A shows a significant increase in both VCAM-1 (*p* < 0.0001) and ICAM-1 (*p* < 0.0001) membrane exposure following treatment with TNF-α, in both cell populations, compared to basal conditions. If anything, there was a trend of higher expression of both molecules in GD-HUVECs compared to C-HUVECs. Noticeably, 24 h AM incubation produced a significant decrease in VCAM-1 and ICAM-1 protein exposure to the cell cytosolic membrane promoted by TNF-α, in both C- (VCAM-1 *p* < 0.0001 and ICAM-1 *p* < 0.05, Figure 2A) and GD-HUVEC populations (VCAM-1 *p* < 0.0001 and ICAM-1 *p* < 0.001, Figure 2A).

To measure the real impact on monocyte adhesion of the variation caused by AM on the expression of the endothelial adhesion molecules, the binding of U937 monocyte cell line to AM-treated C- and GD-HUVECs was investigated. Of note, the representative high-power field pictures showed that AM treatment induces a reduction in the monocyte adhesion rate that was very evident when comparing TNF-α-treated cells to the same treatment supplemented with AM (Figure 2B). Additionally, AM caused an improvement in cells’ morphology compared to cells under basal conditions (Figure 2B). The quantification of adhered monocytes in pictures showed that, under basal conditions, the monocyte adhesion rate was higher in GD-HUVECs than that in C-HUVECs (*p* < 0.05), indicating the GD-HUVEC pro-inflammatory phenotype. Next, a trend of reduction in monocyte–endothelial cell interaction was observed following AM incubation in both cell populations, especially in GD-HUVECs (*p* < 0.05). As expected, 16 h of TNF-α stimulation significantly enhanced the number of adhered monocytes both in C- and GD-HUVEC cultures, as compared to basal (*p* < 0.0001). Strikingly, 24 h of AM incubation, following TNF-α pre-treatment, induced a significant reduction of monocyte adhesion in both C- and GD-HUVECs (*p* < 0.01 and *p* < 0.0001, respectively) (Figure 2C).

Altogether, these data indicate that AM produces a powerful effect on the attenuation of TNF-α expression of endothelial adhesion molecule genes. This is reflected in the number of adhesion molecules detected at the cytosolic membrane of the cells that causes strong differences in the adhesion of monocytes.

### Amniotic Membrane Attenuates the Phosphorylation of NF-kB and Its Nuclear Translocation in Response to TNF-α

**FIGURE 3:**
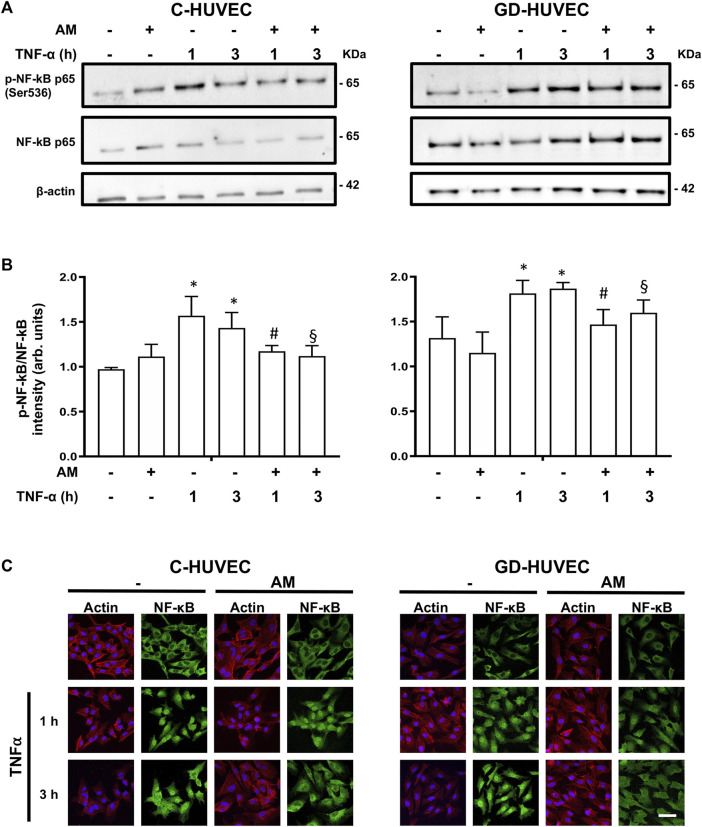
Amniotic membrane attenuates TNF-⍺-induced NF-κB phosphorylation and nuclear translocation. Sub-confluent C-HUVECs and GD-HUVECs were serum-starved, treated with AM for 24h, then TNF-α was added at 1 and 3 h. **(A)** Protein extract was analyzed for WB for the mentioned antibodies. **(B)** Histograms show Western blot quantification of NF-κB phosphorylation experiments (*n* = 3); *p< 0.05 vs basal; #*p*< 0.05 vs TNF-α 1 h; §*p*< 0.05 vs TNF-α 3 h. **(C)** Samples were immunostained with a specific antibody against NF-κB and co-staining with Hoechst-33258 and phalloidin to reveal nuclei and he actin cytoskeleton, respectively. This experiment was repeated at least three times. Representative images are shown.

NF-kB, a major mediator of vascular inflammation, is involved in the expression of critical molecules participating in the adhesion of monocytes to HUVECs (Di Tomo et al., 2012). To further investigate the possible link between the anti-inflammatory role of AM and the attenuation of TNF-α signaling, we looked at NF-kB phosphorylation during TNF-α stimulation. As expected, TNF-α treatment produced a neat increase in NF-kB phosphorylation that was clearly attenuated when cells had been pretreated with AM for 24 h (Figures 3A,B). Moreover, stimulation of both C-HUVECs and GD-HUVECs with TNF-α caused a very evident NF-kB nuclear translocation at 1 and 3 hours (Figure 3C). Consistently with phosphorylation data, 24 hours of preincubation with AM significantly decreased the nuclear translocation of NF-kB in response to TNF-α stimulation in both C- and GD-HUVECs, this was especially evident at time 3 hours (Figure 3C).

These data suggest that the effect seen on the expression of the genes and further protein localization at the cytosolic membrane of HUVECs could be related to the differential phosphorylation and translocation of NF-kB in response to TNF-α depending on whether the cells had been previously treated with AM.

### Amniotic Membrane Improves NO Bioavailability

**FIGURE 4:**
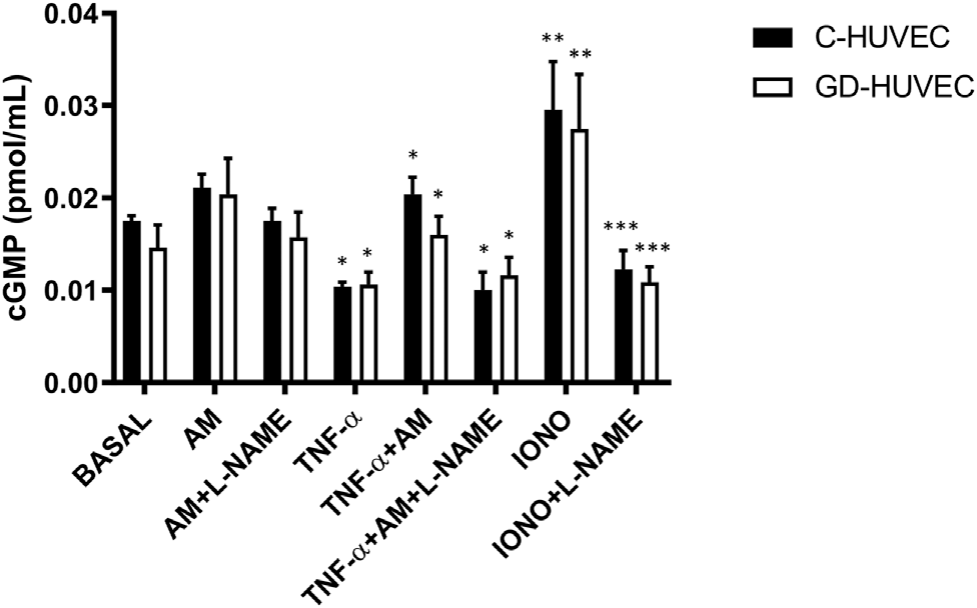
Effect of AM on intracellular cGMP levels measured by ELISA in untreated (basal) and TNF-α-stimulated C- and GD-HUVECs after preincubation for 24 h with AM. Ionomycin (2 μM, 24 h) was used as a positive control. l-NAME (1 mmol/L) was added 45 min before AM and Ionomycin. Data are expressed as pmol/well and results by mean ± SEM, n = 4. ANOVA and Tukey’s multiple comparison test: *p < 0.05 TNF-α vs. basal; TNF-α+AM vs. TNF-α; TNF-α +AM+L-NAME vs. TNF-α+AM; **p < 0.01 IONO vs. basal; ***p < 0.001 IONO+L-NAME vs. IONO.

Nitric oxide deficiency has been proven as an important mechanism associated with vascular inflammation and it is responsible for poor healing in DFU patients (Ahmad 2016). Thus, we evaluated whether AM may modulate NO bioavailability in HUVECs by means of measurement of cGMP levels. As shown in Figure 4, following 24 h of AM treatment, basal cGMP levels slightly increased in both C- and GD-HUVECs. As expected, when both cell cultures were pre-treated with TNF-α, cGMP levels decreased (*p* < 0.05) and, interestingly, the NO bioavailability was significantly increased by AM incubation. Of note, preincubation with l-NAME, the inhibitor of constitutive isoforms of nitric oxide synthase (NOS), produced a clear inhibition of both ionomycin (positive control) and AM stimulating effect, which was completely abolished by l-NAME preincubation (Figure 4). Altogether these data suggest that AM was able to counteract the effect of TNF-α increasing NO bioavailability through eNOS enzymatic activity.

### Amniotic Membrane Stimulates Matrigel Tube Formation in GD-HUVECs

**FIGURE 5:**
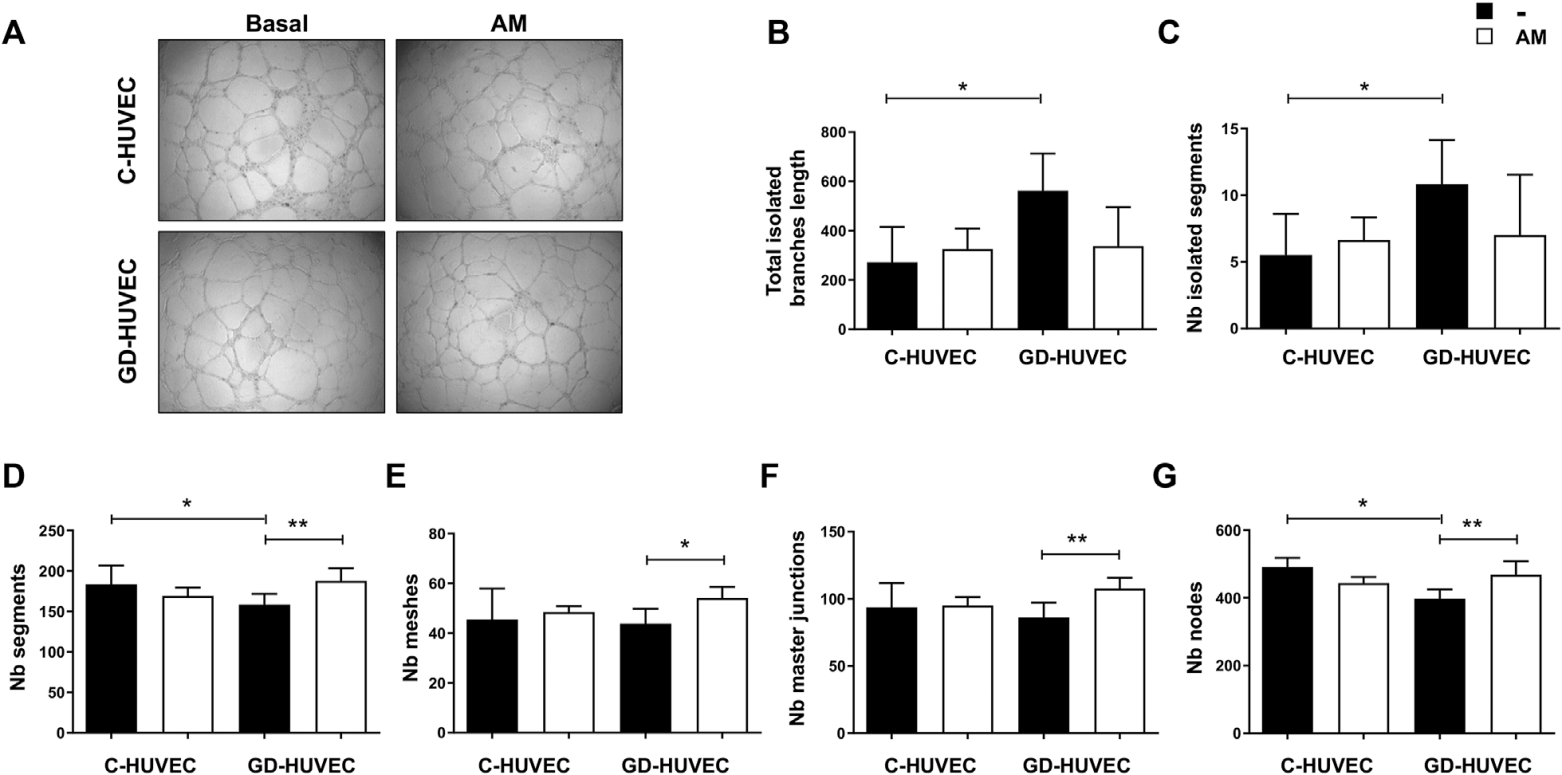
Effect of AM on tube-like structure formation capacity on Matrigel. **(A)** Representative images of C- and GD-HUVECs for both experimental conditions. **(B–G)** Different angiogenic parameters analyzed in C- and GD-HUVECs under basal conditions and after 6 h of AM incubation. Each value is expressed as mean ± SD using three different cellular strains for C-HUVECs and three different cellular strains for GD-HUVECs (*n* = 3). ANOVA and Tukey’s multiple comparison test: *p < 0.05, **p < 0.01.

HUVECs are the most used cells to perform the Matrigel tube formation assay, a test of powerful utility to screen the angiogenic activity of vascular endothelial cells *in vitro*. Since diabetic foot ulcers are characterized by impaired blood vessel formation, the AM capacity in improving endothelial cell network-like structure generation, using the Matrigel tube formation assay, was evaluated. To validate the AM effect, several angiogenic parameters were analyzed following vessel formation as shown in the representative images in Figure 5A.

The effect of AM on C- and GD-HUVEC tubulation is shown in Figure 5A. To better understand the consequences of AM application, several parameters were tested, measured, and their values are represented (Figures 5B–G). Under basal conditions, the total isolated branch length (Figure 5B) and the number of isolated segments (Figure 5C) were increased in GD-HUVECs compared to control ones (*p* < 0.05), thus supporting the hypothesis of impaired angiogenesis in GD-HUVECs. Of note, the AM incubation induced a trend in the reduction of isolated branch length and isolated segments in GD-cells, therefore, indicating the potential of AM in improving the network formation (Figures 5B,C).

For all the other parameters analyzed (Figures 5D,E), under the basal conditions, GD-HUVECs showed a reduced level, thus further supporting the hypothesis of reduced angiogenesis in diabetic cells. Importantly, the AM incubation enhanced the number of segments in GD-HUVECs (*p* < 0.01, Figure 5D), as well as the number of meshes was improved (*p* < 0.05, Figure 5E). Again, the number of master junctions and the number of nodes (Figures 5F,G) increased after AM exposure in GD-HUVECs compared to the basal condition (*p* < 0.01), thus indicating an improvement in network interconnections. On the contrary, C-HUVECs did not show any significant variation in the parameters analyzed.

Overall, the analysis performed here showed impaired vessel formation in GD-HUVECs as demonstrated by the low rate of interconnections and all the analyzed elements were improved following 6 h of AM incubation.

### AM Stimulates Cell Migration in GD-HUVECs

AM has a positive effect on the cell migration of different cell types that is faithfully reflected in the tangible rearrangement of migration machinery (Alcaraz et al., 2015; Bernabé-García et al., 2017; Ruiz-Cañada et al., 2018). Indeed, AM is able to induce the overexpression and activation of very important proteins for cell migration, such as c-Jun (Castellanos et al., 2017).

Many endothelial cell functions are mediated by the ability of endothelial cells to migrate, either to the site of new blood vessels or to repair a vessel wound (Lamalice et al., 2007). In angiogenesis, which is considered to be particularly important for vascular formation in adults, the migration of vascular endothelial cells is deeply involved (Serini et al., 2003; Folkman 2007; Clapp et al., 2009).

**FIGURE 6:**
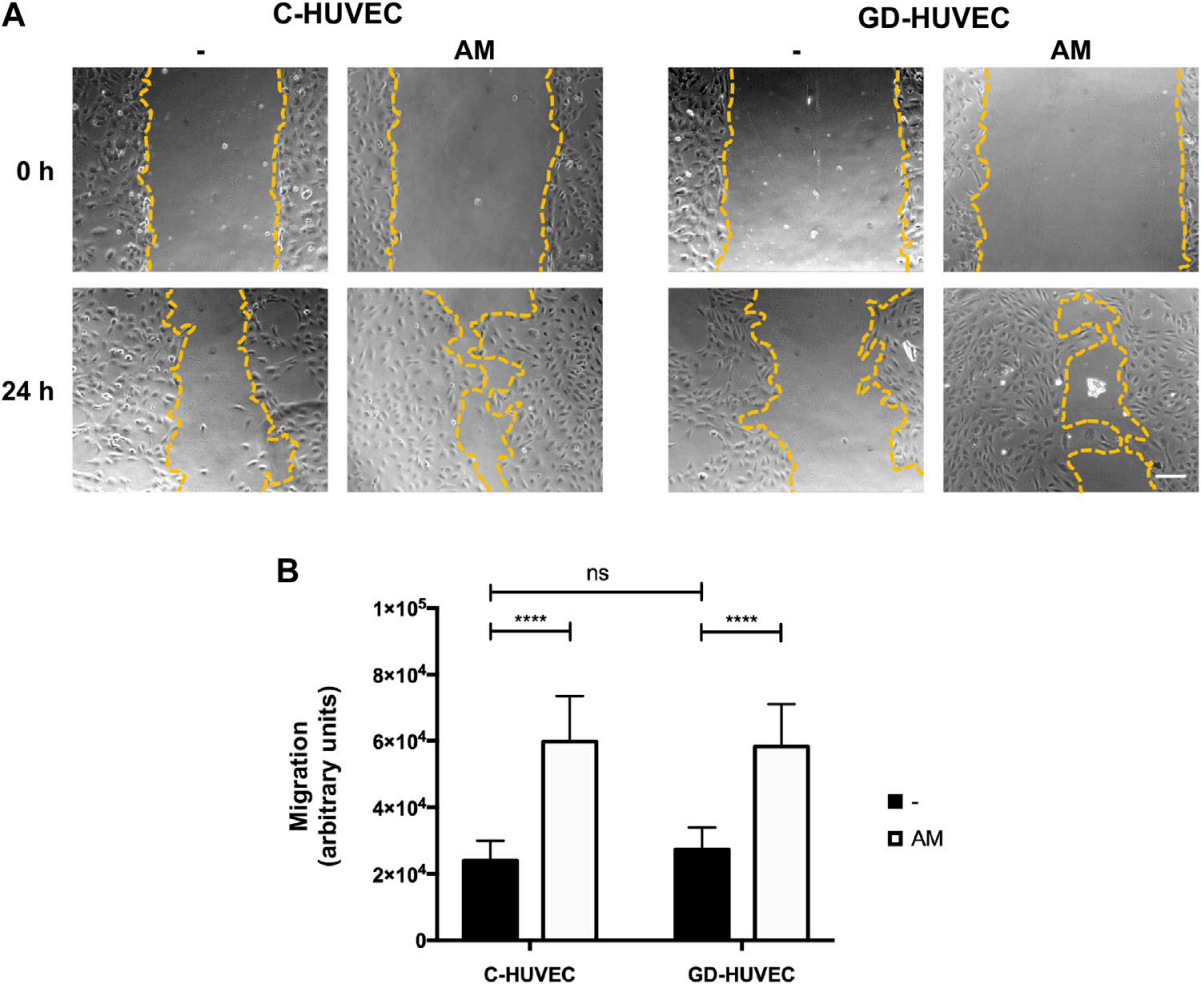
AM induces cell migration in C- and GD-HUVECs. **(A)** HUVECs were wounded and allowed to migrate for the indicated time in the presence or absence of AM. Representative scratch assay from each experimental condition are shown. The wound edges are outlined in yellow. **(B)** Quantification of the difference of migration between 0 and 24 h. ANOVA and Tukey’s multiple comparison test: ****p < 0.0001.

**FIGURE 7:**
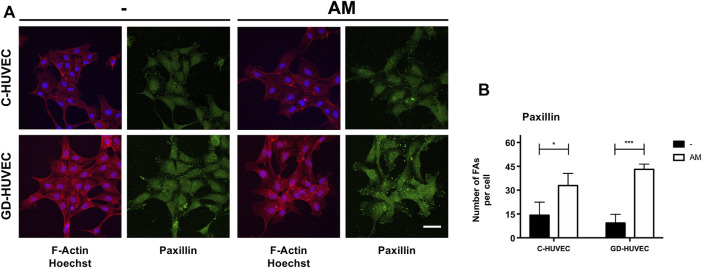
AM induces an active remodeling of focal adhesions (FAs). **(A)** Sub-confluent C-HUVECs and GD-HUVECs were serum-starved cultured for 24 h in presence or absence of AM. Then, samples were immunostained with a specific antibody against paxillin and co-stained with Hoechst-33258 and phalloidin to reveal nuclei and the actin cytoskeleton, respectively. All images were acquired using a confocal microscope and processed using ZEN software. These experiments were repeated at least three times. Representative images are shown. Bar represents 50 µm. **(B)** Focal adhesion (FA) quantification of Paxillin. All images were analyzed through ImageJ software. The plots represent the mean of several technical replicates of three C-HUVEC and three GD-HUVEC strains (*n* = 3). The number of FAs as the region of interests (ROIs) was selected and measured. *p < 0.05 and ***p < 0.001.

Here, first, we measured cell migration on C- and GD-HUVECs using the wound healing scratch assay (Figure 6). Following 24 h, the wounded area was examined by phase-contrast microscopy. Interestingly, the amniotic membrane significantly enhanced cell migration in both C- and GD-HUVECs (Figure 6B, *p* < 0.0001). Then, we studied the subcellular localization of paxillin, a protein involved in the formation of focal adhesion that allows the monitorization of the formation and disassembly of cell adhesions (Mayor and Etienne-Manneville, 2016). When the cells migrate, paxillin-marked focal adhesions reduce their size and become more abundant, suggesting a rapid turnover of these structures to favor cell migration (Mayor and Etienne-Manneville, 2016). The examination of C- and GD-HUVECs treated with AM showed a clear increase in the number of focal adhesions (FAs) with an apparent lower size (Figures 7A,B and Supplementary Figure S2).

These data would be coherent with an increment of cell motility that may explain the better tubulation experienced by GD-HUVECs in the presence of AM.

## Discussion

In the present study, we investigated the potential anti-inflammatory and pro-angiogenic role of AM by using the *in vitro* model of endothelial cells derived from the umbilical cord vein of gestational diabetes women (GD-HUVECs), a precious cellular model of endothelial dysfunction taking place during *in vivo* hyperglycemia (Di Tomo et al., 2021). This study was based on the evidence that the progression of a non-healing phenotype in DFUs is closely linked to increased inflammation, reduced cell migration, and poor vascular networks (Okonkwo and Dipietro, 2017).

The use of placental-based tissues is proposed to aid in healing through the reduction of inflammation, the enhancement of cell migration into the wound environment, the stimulation of cell proliferation, and the improvement of angiogenesis (Pipino et al., 2013; Castellanos et al., 2017).

Chronic wounds fail to proceed to closure for various reasons, impaired vascularization is one of them, and that causes a long-lasting inflammatory process, alterations in the proliferative phase, and cell senescence (Castellanos et al., 2017). The benefits of AM in human therapy have been well established either by using it as a whole dressing or by using AM extracts accompanied by any matrix vehicle (Parolini and Caruso, 2011; Murphy et al., 2017). Apart from its immunomodulatory/anti-inflammatory effects, AM exhibits anti-bacterial properties and low immunogenicity (Parolini et al., 2009). Several clinical studies have used AM for the curing of burn injuries, skin wounds, DFU, chronic leg ulcers, and ocular surface reconstruction (Ornella Parolini et al., 2009; Insausti et al., 2010). In a direct collaboration with the diabetic foot ulcer unit of the Hospital Clínico Universitario Virgen de la Arrixaca (Murcia, Spain), we followed the evolution of 11 patients with DFU that had been treated with AM. A very successful outcome of the treatment shows the wound healing acceleration and a positive evolution of different DFUs upon AM treatment (Valiente et al., 2018).

In 2017, Laurent et al. (2017) , performed a meta-analysis which included different clinical trials to test AM effects on diabetic patients’ foot ulcers. The results showed that the amniotic membrane treatment combined with the standard of care (SOC) is preferable, compared to SOC alone, in terms of efficiency and timing, thus highlighting all the aforementioned properties of AM (Laurent et al., 2017). However, the specific regulatory mechanisms behind the AM efficacy in diabetic wounds are not completely understood (Koob et al., 2014).

The management of inflammation is crucial in resolving chronic wounds. Here, we found that the level of several genes involved in inflammation decreased upon AM treatment. Of note, the decreased level of *VCAM-1* and *ICAM-1* and even more their reduced membrane exposure following AM addition is of particular interest since it is known that these adhesion molecules are found elevated in non-healing DFUs (Kolluru et al., 2012).

To further investigate the amniotic membrane’s anti-inflammatory activity, we determined the monocyte endothelial cell interaction rate, which plays a crucial role in the formation of atheroma. Indeed, the adhesion of circulating monocytes to the endothelial cells, mediated by the interaction with adhesion molecules, is considered one of the earliest events in atherosclerosis (Čejková et al., 2016). After attaching to the endothelium, monocytes subsequently invade the vascular wall and play a central role in triggering the inflammation. Moreover, inflammation has been shown to delay wound healing, therefore, the inhibition of monocyte adhesion to the vessel results in reduced secondary damages, such as reduced inflammation, being beneficial to the wound healing (Cowin et al., 2021).

Here, we found that stimulation with a low concentration of TNF-α induced an increase in the monocyte–endothelial cell interaction that was further enhanced in GD-HUVECs. The presence of AM reduced the amount of adhered monocyte in both cell populations; however, it was especially significative in GD-HUVECs.

NF-κB plays a crucial role in the expression of proinflammatory molecules such as cytokines, chemokine, and adhesion molecules (Dinh and Lewis, 2019) and several studies have demonstrated its involvement in metabolic disorders and atherosclerosis (Di Tomo et al., 2012), phenomena that could be prevented or ameliorated by several pharmacological or natural approaches (Di Pietro et al., 2020). Interestingly, here we found that, following TNF-α-stimulation, AM pre-treatment of HUVECs significantly reduced NF-κB phosphorylation and nuclear translocation in both control cells and GD-HUVECs, thus further confirming the capability of AM in reducing inflammation.

Then, we evaluated the potential role of AM in modulating nitric oxide bioavailability which is involved in the modulation of the NF-κB pathway and thus in the vascular homeostasis balance. Of note, in GD-HUVECs chronically exposed to high glucose and inflammation, impaired nitric oxide synthase (eNOS) activity and enhanced reactive oxygen species (ROS) production are responsible for reduced bioavailability of nitric oxide thus leading to pro-atherogenic alterations (Pandolfi and De Filippis, 2007). Interestingly, in our cellular model, these effects were associated with an ability of AM to enhance NO bioavailability thus protecting HUVECs from TNF-α-induced inflammation.

On the other hand, the AM angiogenic effect is rather controversial. Indeed, AM shows anti-angiogenic features in ophthalmology (Jirsova and Jones, 2017) and pro-angiogenic properties when used to treat skin wounds and DFUs. This is probably due to the simultaneous presence of both pro- and anti-angiogenic molecules in AM. Therefore, the specific AM effect changes among conditions and target tissues (Niknejad and Yazdanpanah, 2014).

In the present study, the AM capacity to improve endothelial cells’ migration and vessel formation was assessed. Supported by these observations, we focused our study on the impaired angiogenic phase that occurs in diabetic wounds. Therefore, the AM role in vessel formation was investigated using the Matrigel tube formation assay. It is worthy of note that the analysis of some angiogenic parameters such as the number of isolated segments and length of isolated branches shows that GD-HUVECs induced the formation of poor interconnected tubes compared to C-HUVECs. On the contrary, as compared to C-HUVECs, the number of segments, meshes, and master junctions and the number of nodes were reduced in GD-HUVECs, further assessing impaired angiogenesis in these cells. Additionally, GD-HUVEC data clearly showed basal impaired angiogenesis when compared to control cells. This further supports the idea that chronic hyperglycemia, *per se*, may strongly reduce the angiogenesis in GD-HUVECs. Of note, all these parameters were improved in GD-HUVECs following incubation with AM, leading to a better vascular network formation. Moreover, it should be noticed that from the data obtained by angiogenic analysis, most of the parameters in C-HUVECs were not ameliorated following AM incubation. Our explanation to these findings is that C-HUVECs already possess an intrinsic sufficient capability to achieve a full network. On the contrary, the impaired networking capacity of GD-HUVECs, was improved after incubation with AM, thus reaching the same levels of C-HUVECs. Collectively, these observations are consistent with our previous findings and may explain some of the reported beneficial effects of AM *in vivo* when applied to DFUs (Valiente et al., 2018)*.* Also, these results are suggestive of the induction of a series of events leading to a better angiogenesis produced by AM in DFUs*.*

Some interesting studies reported the capability of AM in improving the migration of keratinocytes (Ruiz-Cañada et al., 2018), however, only some studies investigated the effect of AM on HUVECs migration (Koob et al., 2014; Duan-Arnold et al., 2015) but never in GD-HUVECs. When tested, AM was beneficial for the migration of both C- and GD-HUVECs. However, to our surprise, we could not detect differences between the type of cells in the migration either before or after AM stimulation.

The migrating effect of AM can also be evaluated by looking at the quality and quantity of focal adhesions (FAs). Indeed, the effect of AM on HaCaT and Mv1Lu migrating cells was a reduction in size and increment in numbers of FAs at the time of cell migration (Bernabé-García et al., 2017). This is due to a rapid formation and disassembly of cell adhesion sites that are required, and it is observed in actively migrating cells (Mayor and Etienne-Manneville, 2016). Cell migration is coordinated by a complex of proteins that localizes to sites of cell–matrix interaction, the FAs (Critchley 2000; Geiger et al., 2001). Paxillin is a key cytosolic protein that coordinates the binding of integrin cell attachment sites to the actin cytoskeleton (López-Colomé et al., 2017). Paxillin also takes part in FAs, and it is essential to integrate different signals related to cell migration (Huang et al., 2003). In HaCaT cells, AM induces paxillin phosphorylation at Ser-178 and also produces an intense remodeling of FA paxillin staining, which are critical for cell migration (Bernabé-García et al., 2017). In the presence of AM, a clear remodeling of focal adhesions was observed when those were revealed by paxillin staining. Hence, altogether, the fact that this migration intensively related protein gets remodeled in the presence of AM is suggestive that a migration process is stimulated by the presence of AM. The strong FA remodeling effect of AM, measured as the number of paxillin structures, was equally effective in C- and GD-HUVECs, in agreement with the data of migration for both types of cells. To this end, however, whether AM promoted migration could add an extra advantage in resolving DFUs, should be deeply investigated either in *in vivo* models or patients. Altogether, this work confirms that the use of AM is a desirable strategy in DFUs and it provides further insights to elucidate the therapeutic effects of AM observed in preclinical studies.

## Conclusion

**FIGURE 8:**
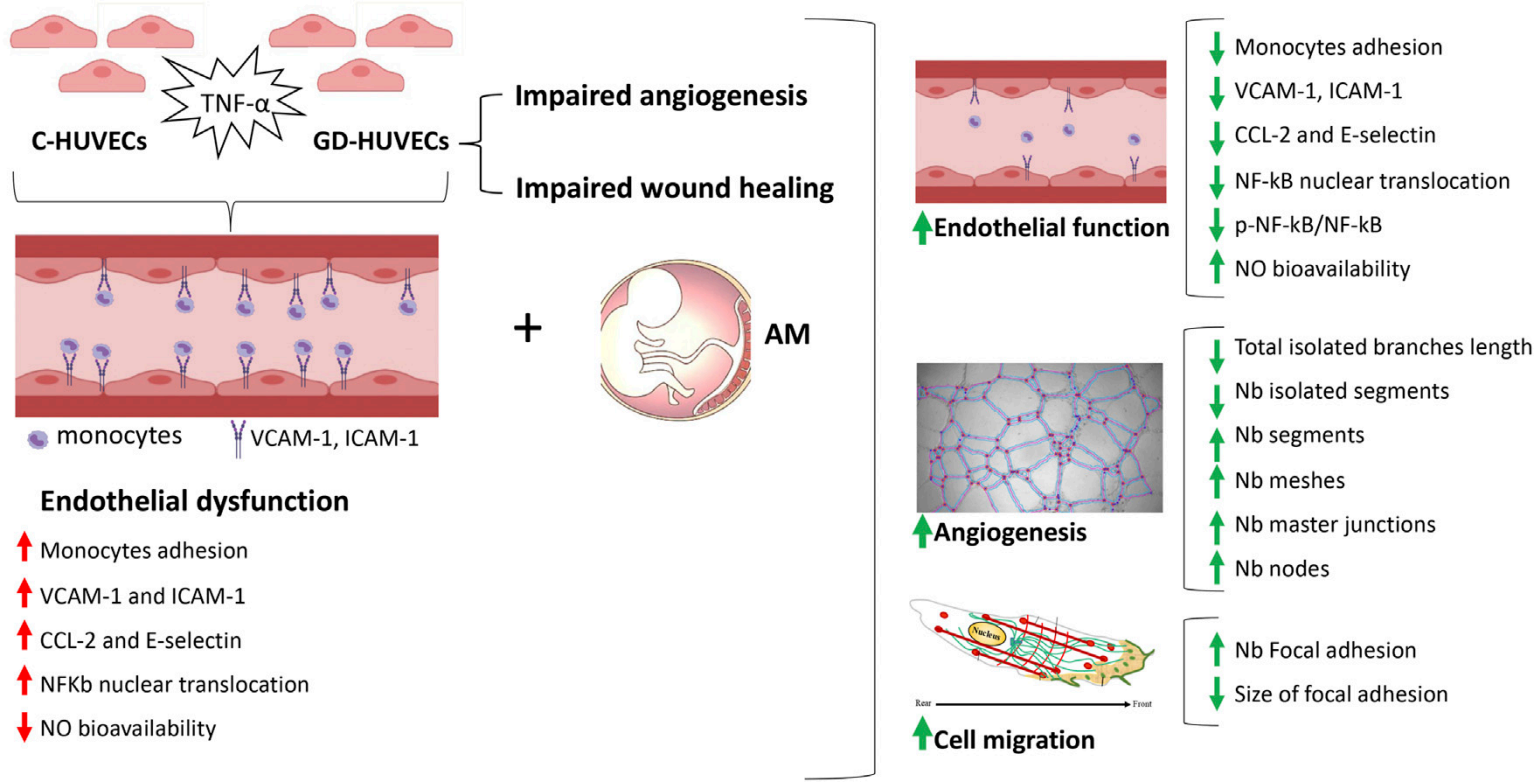
Schematic representation of the main findings from the study. Gestational diabetes endothelial cells (GD-HUVECs) have shown increased inflammation leading to endothelial dysfunction as well as impaired angiogenesis and wound healing. By using this cellular model that reproduces a typical diabetic foot ulcer endothelium, the amniotic membrane (AM) was able to restore endothelial function, enhance angiogenesis, and improve wound healing.

In this study, we shed light on the mechanisms related to the AM capability to heal inflammation and improve angiogenesis in endothelial cells obtained from the umbilical cord vein of gestational diabetic mothers, supporting preclinical results. Although further research is needed to evaluate how these findings translate to diabetic foot ulcer patients, all together, these results carried out through an innovative *in vitro* cellular model, assess the role of the amniotic membrane to induce a significant reduction of the inflammation typical of chronic diabetic foot ulcers and to improve endothelial cell vascular network formation (Figure 8).

## Data Availability

The original contributions presented in the study are included in the article/Supplementary Materials, further inquiries can be directed to the corresponding authors.

## References

[B1] Abacı A. Oguzhan A. Kahraman S. Eryol N. K. Unal S. Arınc H. (1999). Effect of Diabetes Mellitus on Formation of Coronary Collateral Vessels. Circulation 99 (17), 2239–2242. 10.1161/01.CIR.99.17.2239 10226087

[B2] Ahmad J. (2016). The Diabetic Foot. Diabetes & Metabolic Syndrome Clin. Res. Rev. 10 (1), 48–60. 10.1016/j.dsx.2015.04.002 26072202

[B3] Alcaraz A. Mrowiec A. Insausti C. L. Bernabé-García Á. García-Vizcaíno E. M. López-Martínez M. C. (2015). Amniotic Membrane Modifies the Genetic Program Induced by TGFß, Stimulating Keratinocyte Proliferation and Migration in Chronic Wounds. PLoS ONE 10 (8), e0135324. 10.1371/journal.pone.0135324 26284363 PMC4540284

[B4] Bandyk D. F. (2018). The Diabetic Foot: Pathophysiology, Evaluation, and Treatment. Seminars Vasc. Surg. 31 (2–4), 43–48. 10.1053/j.semvascsurg.2019.02.001 30876640

[B5] Bernabé-García Á. Liarte S. Moraleda J. M. Castellanos G. Nicolás F. J. (2017). Amniotic Membrane Promotes Focal Adhesion Remodeling to Stimulate Cell Migration. Sci. Rep. 7 (1), 1–12. 10.1038/s41598-017-15509-z 29127427 PMC5681678

[B6] Boykin J. V. (2010). Wound Nitric Oxide Bioactivity. J. Wound, Ostomy & Cont. Nurs. 37 (1), 25–32. 10.1097/won.0b013e3181c68b61 20075688

[B7] Castellanos G. Bernabé-García Á. Moraleda J. M. Nicolás F. J. (2017). Amniotic Membrane Application for the Healing of Chronic Wounds and Ulcers. Placenta 59, 146–153. 10.1016/j.placenta.2017.04.005 28413063

[B8] Čejková S. Králová Lesná I. Poledne R. (2016). Monocyte Adhesion to the Endothelium Is an Initial Stage of Atherosclerosis Development. Cor Vasa 58 (4), e419–e425. 10.1016/j.crvasa.2015.08.002

[B9] Clapp C. Thebault S. Jeziorski M. C. Martínez De La Escalera G. (2009). Peptide Hormone Regulation of Angiogenesis. Physiol. Rev. 89, 1177–1215. 10.1152/physrev.00024.2009 19789380

[B10] Cowin A. J. Bayat A. Murray R. Z. Kopecki Z. (2021). Editorial: Inflammation in Healing and Regeneration of Cutaneous Wounds. Front. Immunol. 12 (November), 10–12. 10.3389/fimmu.2021.806687 PMC8639488 34868091

[B11] Critchley D. R. (2000). Focal Adhesions - The Cytoskeletal Connection. Curr. Opin. Cell Biol. 12 (1), 133–139. 10.1016/s0955-0674(99)00067-8 10679361

[B12] Di Fulvio P. Pandolfi A. Formoso G. Di Silvestre S. Di Tomo P. Giardinelli A. (2014). Features of Endothelial Dysfunction in Umbilical Cord Vessels of Women with Gestational Diabetes. Nutr. Metabolism Cardiovasc. Dis. 24 (12), 1337–1345. 10.1016/j.numecd.2014.06.005 25438716

[B13] Di Pietro N. Baldassarre M. P. A. Cichelli A. Pandolfi A. Formoso G. Pipino C. (2020). Role of Polyphenols and Carotenoids in Endothelial Dysfunction: An Overview from Classic to Innovative Biomarkers. Oxidative Med. Cell. Longev. 2020, 1–19. 10.1155/2020/6381380 PMC7593735 33133348

[B14] Di Tomo P. Alessio N. Falone S. Pietrangelo L. Lanuti P. Cordone V. (2021). Endothelial Cells from Umbilical Cord of Women Affected by Gestational Diabetes: A Suitable *In Vitro* Model to Study Mechanisms of Early Vascular Senescence in Diabetes. FASEB J. 35, e21662. 10.1096/fj.202002072RR 34046935 PMC12315945

[B15] Di Tomo P. Canali R. Ciavardelli D. Di Silvestre S. De Marco A. Giardinelli A. (2012). β-Carotene and Lycopene Affect Endothelial Response to TNF-α Reducing Nitro-Oxidative Stress and Interaction with Monocytes. Mol. Nutr. Food Res. 56 (2), 217–227. 10.1002/mnfr.201100500 22162208

[B16] Di Tomo P. Di Silvestre S. Cordone V. G. P. Giardinelli A. Faricelli B. Pipino C. (2015). Centella Asiatica and Lipoic Acid, or a Combination Thereof, Inhibit Monocyte Adhesion to Endothelial Cells from Umbilical Cords of Gestational Diabetic Women. Nutr. Metabolism Cardiovasc. Dis. 25 (7), 659–666. 10.1016/j.numecd.2015.04.002 26026207

[B17] Dinh T. Lewis C. (2019). Amnion Applications in the Foot and Ankle. Clin. Podiatric Med. Surg. 36 (4), 563–576. 10.1016/j.cpm.2019.06.004 31466568

[B18] Doucette M. Payne K. M. Lough W. Beck A. Wayment K. Huffman J. (2020). Early Advanced Therapy for Diabetic Foot Ulcers in High Amputation Risk Veterans: A Cohort Study. Int. J. Low. Extrem. Wounds 21, 111–119. Wounds 1534734620: 1–9. 10.1177/1534734620928151 32567415 PMC7752820

[B19] Duan-Arnold Y. Uveges T. E. Gyurdieva A. Johnson A. Danilkovitch A. (2015). Angiogenic Potential of Cryopreserved Amniotic Membrane Is Enhanced Through Retention of All Tissue Components in Their Native State. Adv. Wound Care 4 (9), 513–522. 10.1089/wound.2015.0638 PMC4528990 26339531

[B20] Eelen G. De Zeeuw P. Simons M. Carmeliet P. (2015). Endothelial Cell Metabolism in Normal and Diseased Vasculature. Circ. Res. 116 (7), 1231–1244. 10.1161/CIRCRESAHA.116.302855 25814684 PMC4380230

[B21] Efron D. T. Most D. Barbul A. (2000). Role of Nitric Oxide in Wound Healing. Curr. Opin. Clin. Nutr. Metabolic Care 3, 197–204. 10.1097/00075197-200005000-00006 10871235

[B22] Folkman J. (2007). Angiogenesis: An Organizing Principle for Drug Discovery? Nat. Rev. Drug Discov. 6, 273–286. 10.1038/nrd2115 17396134

[B23] Geiger B. Bershadsky A. Pankov R. Yamada K. M. (2001). Transmembrane Crosstalk Between the Extracellular Matrix and the Cytoskeleton. Nat. Rev. Mol. Cell Biol. 2 (11), 793–805. 10.1038/35099066 11715046

[B24] Huang C. Rajfur Z. Borchers C. Schaller M. D. Jacobson. K. (2003). JNK Phosphorylates Paxillin and Regulates Cell Migration. Nature 424 (6945), 219–223. 10.1038/nature01745 12853963

[B25] Insausti C. L. Alcaraz A. García-Vizcaíno E. M. Mrowiec A. López-Martínez M. C. Blanquer M. (2010). Amniotic Membrane Induces Epithelialization in Massive Posttraumatic Wounds. Wound Repair Regen. 18 (4), 368–377. 10.1111/j.1524-475X.2010.00604.x 20636551

[B26] Jirsova K. Jones G. L. A. (2017). Amniotic Membrane in Ophthalmology: Properties, Preparation, Storage and Indications for Grafting-A Review. Cell Tissue Bank. 18 (2), 193–204. 10.1007/s10561-017-9618-5 28255771

[B27] Kolluru G. K. Bir S. C. Kevil C. G. (2012). Endothelial Dysfunction and Diabetes: Effects on Angiogenesis, Vascular Remodeling, and Wound Healing. Int. J. Vasc. Med. 2012, 1–30. 10.1155/2012/918267 PMC3348526 22611498

[B28] Koob T. J. Lim J. J. Massee M. Zabek N. Rennert R. Gurtner G. (2014). Angiogenic Properties of Dehydrated Human Amnion/Chorion Allografts: Therapeutic Potential for Soft Tissue Repair and Regeneration. Int. J. Mol. Sci. 18, 1419. 10.1186/2045-824X-6-10 PMC4016655 24817999

[B29] Lakmal K. Basnayake O. Hettiarachchi D. (2021). Systematic Review on the Rational Use of Amniotic Membrane Allografts in Diabetic Foot Ulcer Treatment. BMC Surg. 21 (87), 1–8. 10.1186/s12893-021-01084-8 33588807 PMC7885244

[B30] Lamalice L. Le Boeuf F. Huot J. (2007). Endothelial Cell Migration During Angiogenesis. Circulation Res. 100, 782–794. 10.1161/01.RES.0000259593.07661.1e 17395884

[B31] Laurent I. Astère M. Wang K. R. Cheng Q.-f. Li Q. F. (2017). Efficacy and Time Sensitivity of Amniotic Membrane Treatment in Patients with Diabetic Foot Ulcers: A Systematic Review and Meta-Analysis. Diabetes Ther. 8, 967–979. 10.1007/s13300-017-0298-8 28895073 PMC5630554

[B32] López-Colomé A. M. Lee-Rivera I. Benavides-Hidalgo R. López E. López E. (2017). Paxillin: A Crossroad in Pathological Cell Migration. J. Hematol. Oncol. 10 (1), 1–15. 10.1186/s13045-017-0418-y 28214467 PMC5316197

[B33] Mayor R. Etienne-Manneville S. (2016). The Front and Rear of Collective Cell Migration. Nat. Rev. Mol. Cell Biol. 17 (2), 97–109. 10.1038/nrm.2015.14 26726037

[B34] Medina-Leyte D. J. Domínguez-Pérez M. Mercado I. Villarreal-Molina M. T. Jacobo-Albavera L. Jacobo-Albavera L. (2020). Use of Human Umbilical Vein Endothelial Cells (HUVEC) as a Model to Study Cardiovascular Disease: A Review. Appl. Sci. 10 (3), 938. 10.3390/app10030938

[B35] Murphy S. V. Skardal A. Song L. Sutton K. Haug R. Mack D. L. (2017). Solubilized Amnion Membrane Hyaluronic Acid Hydrogel Accelerates Full-Thickness Wound Healing. Stem Cells Transl. Med. 6 (11), 2020–2032. 10.1002/sctm.17-0053 28941321 PMC6430059

[B36] Niknejad H. Yazdanpanah G. (2014). Opposing Effect of Amniotic Membrane on Angiogenesis Originating from Amniotic Epithelial Cells. J. Med. Hypotheses Ideas 8 (1), 39–41. 10.1016/j.jmhi.2013.08.002

[B37] Okonkwo U. Dipietro L. (2017). Diabetes and Wound Angiogenesis. Ijms 18 (7), 1419–1515. 10.3390/ijms18071419 PMC5535911 28671607

[B38] Pandolfi A. De Filippis E. A. (2007). Chronic Hyperglicemia and Nitric Oxide Bioavailability Play a Pivotal Role in Pro-Atherogenic Vascular Modifications. Genes Nutr. 2 (2), 195–208. 10.1007/s12263-007-0050-5 18850175 PMC2474951

[B39] Parolini O. Caruso M. (2011). Review: Preclinical Studies on Placenta-Derived Cells and Amniotic Membrane: An Update. Placenta 32, S186–S195. 10.1016/j.placenta.2010.12.016 21251712

[B40] Parolini O. Soncini M. Evangelista M. Schmidt D. (2009). Amniotic Membrane and Amniotic Fluid-Derived Cells: Potential Tools for Regenerative Medicine? Regen. Med. 4 (2), 275–291. 10.2217/17460751.4.2.275 19317646

[B41] Pipino C. Shangaris P. Resca E. Zia S. Deprest J. Sebire N. J. (2013). Placenta as a Reservoir of Stem Cells: An Underutilized Resource? Br. Med. Bull. 105, 43–68. 10.1093/bmb/lds033 23184854

[B42] Ruiz-Canada C. Bernabe-García Á. Liarte S. Insausti C. L. Angosto D. Moraleda J. M. (2017). Amniotic Membrane Stimulates Cell Migration by Modulating Transforming Growth Factor‐β Signalling. J. Tissue Eng. Regen. Med. 12 (3), 808–820. 10.1002/term.2501 28621502

[B43] Ruiz-Canada C. Bernabe-Garcia A. Liarte S. Insausti C. L. Angosto D. Moraleda J. M. (2018). Amniotic Membrane Stimulates Cell Migration by Modulating Transforming Growth Factor‐β Signalling. J. Tissue Eng. Regen. Med. 12 (3), 808–820. 10.1002/term.2501 28621502

[B44] Serini G. Ambrosi D. Giraudo E. Gamba A. Preziosi L. Bussolino F. (2003). Modeling the Early Stages of Vascular Network Assembly. EMBO J. 22 (8), 1771–1779. 10.1093/emboj/cdg176 12682010 PMC154468

[B45] Silini A. R. Di Pietro R. Lang-Olip I. Alviano F. Banerjee A. Basile M. (2020). Perinatal Derivatives: Where Do We Stand? A Roadmap of the Human Placenta and Consensus for Tissue and Cell Nomenclature. Front. Bioeng. Biotechnol. 8 (December), 1–33. 10.3389/fbioe.2020.610544 33392174 PMC7773933

[B46] Tentolouris N. Edmonds M. E. Jude E. B. Vas P. R. J. Manu C. A. Tentolouris A. (2021). Editorial: Understanding Diabetic Foot Disease: Current Status and Emerging Treatment Approaches. Front. Endocrinol. 12 753181. 10.3389/fendo.2021.753181 PMC8481651 34603217

[B47] Tettelbach W. Cazzell S. Reyzelman A. M. Sigal F. Caporusso J. M. Agnew P. S. (2019). A Confirmatory Study on the Efficacy of Dehydrated Human Amnion/Chorion Membrane dHACM Allograft in the Management of Diabetic Foot Ulcers: A Prospective, Multicentre, Randomised, Controlled Study of 110 Patients from 14 Wound Clinics. Int. Wound J. 16, 19–29. 10.1111/iwj.12976 30136445 PMC7379535

[B48] Ucci M. Di Tomo P. Tritschler F. Cordone V. G. P. Lanuti P. Bologna G. (2019). Cordone, Paola Lanuti, Giuseppina Bologna, Sara Di Silvestre, et al Anti-Inflammatory Role of Carotenoids in Endothelial Cells Derived from Umbilical Cord of Women Affected by Gestational Diabetes Mellitus. Oxidative Med. Cell. Longev. 2019, 1–11. 10.1155/2019/8184656 PMC6409051 30918580

[B49] Valiente M. R. Nicolás F. J. García-Hernández A. M. Mora C. F. Blanquer M. Alcaraz P. J. (2018). Cryopreserved Amniotic Membrane in the Treatment of Diabetic Foot Ulcers: A Case Series. J. Wound Care 27 (12), 806–815. 10.12968/jowc.2018.27.12.806 30557111

[B50] Wallace J. L. (2005). Nitric Oxide as a Regulator of Inflammatory Processes. Mem. Inst. Oswaldo Cruz 100 (Suppl. 1), 5–9. 10.1590/S0074-02762005000900002 15962091

[B51] Waltenberger J. Lange J. Kranz A. (2000). Vascular Endothelial Growth Factor-A-Induced Chemotaxis of Monocytes Is Attenuated in Patients with Diabetes Mellitus. Circulation 102 (2), 185–190. 10.1161/01.CIR.102.2.185 10889129

[B52] Zelen C. M. (2013). An Evaluation of Dehydrated Human Amniotic Membrane Allografts in Patients with DFUs. J. Wound Care 22 (7), 347–351. 10.12968/jowc.2013.22.7.347 24159656

