# Supplementary material for: Effect of the Human Amniotic Membrane on the Umbilical Vein Endothelial Cells of Gestational Diabetic Mothers: New Insight on Inflammation and Angiogenesis
Source: Front Bioeng Biotechnol. 2022 Jul 5;10:854845. doi: 10.3389/fbioe.2022.854845 (PMC9294233; doi:10.3389/fbioe.2022.854845)
Supplement: Supplementary file 1 [file DataSheet1.PDF]

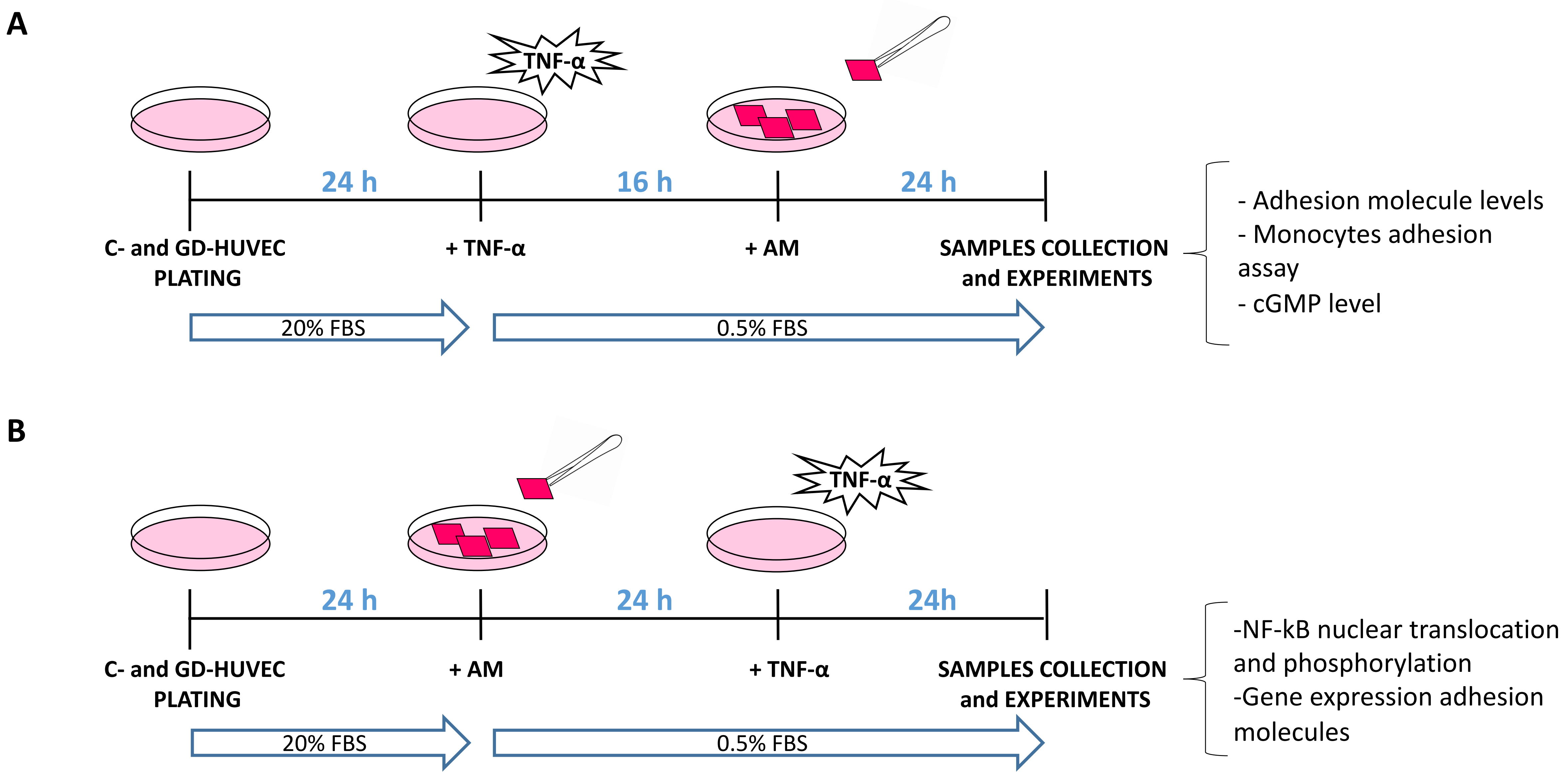

Supplemental Figure 1. Schematic representation of the main protocols used in this work. **(A)** In order to reproduce the inflammatory milieu observed in DFU, C- and GD-HUVECs were stimulated with a low dose of TNF-α (1 ng/ml) for 16 hours and further incubated in the presence of AM for additional 24 hours. **(B)** NF-kB experiments were performed by treating the cells with AM for 24 h and then adding TNF-α at 1h and 3h to evaluate phosphorylation and nuclear translocation.

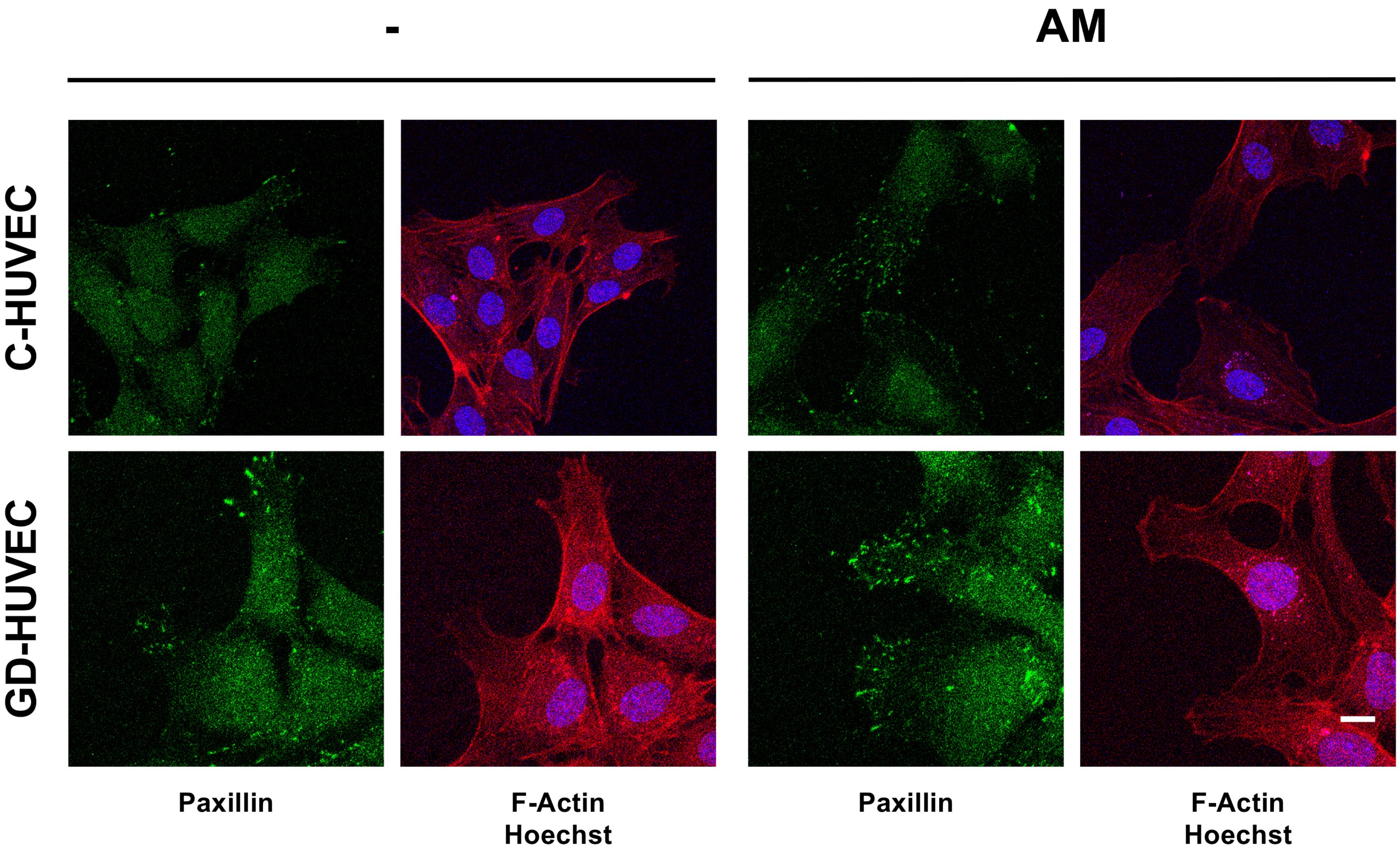

Supplemental Figure 2. Enlargement of pictures in figure 6 showing details of increased number of FA with an apparent lower size. Sub-confluent C-HUVECs and GD-HUVECs were serum-starved cultured for 24 hours period in presence or absence of AM. Samples were immunostained with a specific antibody against Paxillin and co-staining with Hoechst-33258 and phalloidin to reveal nuclei and actin cytoskeleton, respectively. Images were acquired by a confocal microscope and processed by ZEN software. Bar represents 10μm.
